# A Mysterious Fever and Retropharyngeal Edema on a Previously Healthy 10-Year-Old Boy Without Known Exposure to COVID-19

**DOI:** 10.7759/cureus.25373

**Published:** 2022-05-26

**Authors:** Chenxuan Zhou, Mengyao Cheng, Hanyang Hong

**Affiliations:** 1 Pediatrics, Virginia Commonwealth University School of Medicine, Richmond, USA; 2 Department of Nursing, J. Sargeant Reynolds Community College, Richmond, USA; 3 Anesthesiology, Loma Linda University Health Education Consortium, Loma Linda, USA

**Keywords:** persistent fever, echocardiogram, retropharyngeal edema, covid-19, multisystem inflammatory syndrome in children

## Abstract

Multisystem inflammatory syndrome in children (MIS‐C) is considered a late manifestation of COVID-19 infection, and it is a diagnosis of exclusion after ruling out other causes of systemic inflammations. We present a case of MIS-C to highlight the importance of cardiac workup in MIS-C due to frequent cardiac involvement and discuss the possible association between retropharyngeal edema and MIS-C. The case patient is a 10-year-old previously healthy boy who presented with persistent fever, right-side neck pain, and a new rash. The rash was attributed to recent amoxicillin use by his parents. Pertinent workups included elevated inflammatory markers, a benign electrocardiogram test, a negative urine analysis, blood culture, and retropharyngeal edema by computerized tomography. On day four of hospitalization, the patient failed to improve with broad-spectrum antibiotics and became tachycardic. A repeat echocardiogram revealed a decreased ejection fraction with mitral valve regurgitation. The cardiac finding, the skin finding, the persistent fever, and the initial negative workups fulfilled the case criteria for MIS-C. A positive test for anti-SARS-CoV-2 spike protein receptor-binding domain antibodies confirmed the diagnosis, and the patient improved with intravenous immune globulin (IVIG) and steroids. The retropharyngeal edema was thought to be coincidental; however, there seem to be frequent associations between MIS-C and retropharyngeal edema, suggesting that the retropharyngeal edema could be one of the initial manifestations of MIS-C. More study is needed to study the association between retropharyngeal edema and MIS-C and shed light on the diagnosis and medical management of MIS-C.

## Introduction

At the beginning of the COVID-19 pandemic, children were thought to be less susceptible to COVID-19 and develop milder symptoms than adults; however, since April 2020, previously healthy children exposed to COVID-19 infections had previously exposed were rushed to hospitals with cardiogenic shock and Kawasaki disease-like symptoms [1]. More reports of this sequela of the COVID-19 infection in children emerged and started to resonate among the pediatric community [1]. This mysterious syndrome was termed multisystem inflammatory syndrome in children (MIS‐C). Currently, the Centers for Disease Control and Prevention (CDC) defines the criteria for MIS-C as patient below the age of 21 years of age, with laboratory evidence of inflammation, required for hospitalization due to at least two organs involvement, with no plausible alternative diagnosis, and positive laboratory test for COVID-19 infection or exposure within the four weeks prior to the onset of symptoms [2]. We present a case of MIS-C in a pediatric patient with a concurrent retropharyngeal inflammation for three purposes: To highlight to providers that MIS-C should be suspected in pediatric patients presenting with persistent fevers and generalized lethargy even without known exposure to COVID-19, given the current magnitude of the COVID-19 pandemic and the increasing amount of asymptomatic cases; To stress the importance of a bedside echocardiogram for hospitalized pediatric patients with suspicions of MIS-C due to frequent cardiac involvements; To discuss the possible association between retropharyngeal edema and MIS-C.

## Case presentation

**Figure 1:**
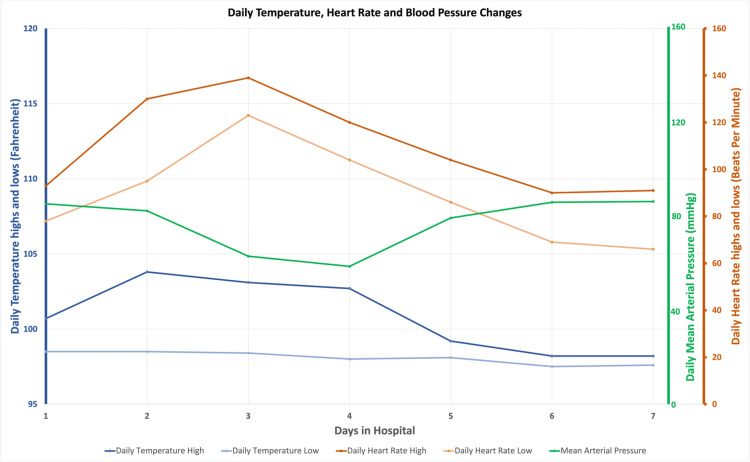
Daily temperature, heart rate, and blood pressure during the hospital stay.

A 10-year-old African American male presented to the Emergency Department (ED) with persistent fever, a diffuse rash, and myalgias. A detailed history of present illness revealed that the patient developed a sore throat and tenderness on the right side of his cervical area under the jaw six days prior to presentation. According to his mother, the tenderness was associated with a fever ranging from 103-104 degrees Fahrenheit, intermittently responding to Acetaminophen and Ibuprofen. The patient was brought to an urgent care three days prior to the ED admission and was tested negative for COVID-19 and Strep throat. However, he was found to have an acute otitis media on the right and was started on oral amoxicillin. The otitis media resolved completely two days after, evidenced by the patient's self-report based on clinical findings and a negative physical examination in the ED. One day before his ED presentation, he developed a diffuse, itchy, maculopapular blanching rash on his arms, legs, back, and chest. The patient's mother attributed the sudden break out of the full-body rash to amoxicillin allergy and stopped the drug. The patient's fever persisted, and the throat pain worsened; together with the newly developed itchy rash, the patient felt extremely ill and was taken to the ED by his mother. A physical exam revealed no signs of nuchal rigidity or meningism. His pharynx was unremarkable, without any obvious edema or uvular deviation. No trismus was noted on exam. Tenderness was elicited on the right anterior/posterior cervical area without adenopathy. A neck CT revealed retropharyngeal fluid with a dimension of 60 x 23 x 8 mm (craniocaudal x transverse x anterior-posterior). Surrounding soft tissues were unremarkable without attenuations and enhancements. The otolaryngologist suggested the fluid was more likely to be edema and less likely to be an abscess. The CT findings are not indicative of drainage or surgery, and the patient was started on clindamycin (600mg IV bid q8h) coverage with close monitoring. Routine screenings and workups performed at the ED were negative for COVID-19, group A Streptococcus, and influenza A/B. Blood culture yielded no growth throughout the hospital course (Table 1). Upon admission, the ECG obtained showed a normal heart rate and sinus rhythm. Per his parents, the patient had not received his COVID-19 vaccine. The other differential was MIS-C, which must be considered an exclusionary diagnosis. However, given that the only organ involved in the inflammatory process was the skin and there was a lack of inflammation evidence in 2 or more organ systems, MIS-C was less likely. From this point on, attention was focused on treating the retropharyngeal fluid collection. A one-time Dexamethasone (4 mg) injection was given to treat the rash and the retropharyngeal fluid collection. Shortly after, the patient's temperature decreased from 103.3 to 99.1 degrees Fahrenheit, and most of the rash resolved. Nevertheless, the patient still felt ill, and his neck pain persisted. The patient was admitted to the pediatric ward. The patient's vital signs (blood pressure, temperature, and heart rate) were documented (Figure 1).

**Table 1:** Respiratory virus panel polymerase chain reaction (PCR) obtained by a nasopharyngeal swab on Day 2 of hospitalization

Adenovirus	Not Detected
Coronavirus 229E	Not Detected
Coronavirus HKU1	Not Detected
Coronavirus NL63	Not Detected
Coronavirus OC43	Not Detected
Novel 2019 Coronavirus	Not Detected
Influenza A	Not Detected
Influenza A 2009 H1	Not Detected
Influenza A H1	Not Detected
Influenza A 2009 H3	Not Detected
Influenza B	Not Detected
Human Metapneumovirus	Not Detected
Human Rhino/entero virus	Not Detected
Parainfluenza 1	Not Detected
Parainfluenza 2	Not Detected
Parainfluenza 3	Not Detected
Parainfluenza 4	Not Detected
Respiratory Syncytial virus	Not Detected
Bordetella pertussis	Not Detected
Chlamydophila Pneumoniae	Not Detected
Mycoplasma Pneumoniae	Not Detected

On day 2 of the patient's hospital course, his temperature was hovering around 101 degrees Fahrenheit despite clindamycin coverage (Figure 1). A respiratory virus panel was conducted due to a suspicion of a viral origin for the retropharyngeal fluid collection (Table 1), but the panel came back negative for all viruses. A routine ECG revealed sinus tachycardia (Figure 1). On day 3, a decision was made to switch from clindamycin (600mg IV bid q8h) to Ceftriaxone (2000mg IV q24h) and Vancomycin (750mg IV q8h) in case the culprit bacteria causing the retropharyngeal edema was clindamycin resistant Methicillin-resistant Staphylococcus aureus (MRSA) or gram-negative pathogens. The etiology of the retropharyngeal edema was unclear but was thought to be related to the moving vehicle accident three weeks ago. The patient's temperature seemed to improve after switching antibiotic coverage; however, the patient spiked a fever again at night. His heart rate remained elevated on the telemetry at 130s beats per minute (Figure 1). Urine analysis and blood culture were obtained during the evening shift to find the source of the persistent fever. The urine analysis returned unremarkably, and the blood culture remained no-growth-to-date throughout his hospital course (Table 2).

**Table 2:** Pertinent laboratory findings during the case patient’s hospitalization WBC: white blood count; RBC: red blood cell; Hgb: hemoglobin; Hct: hematocrit; MCV: mean corpuscular volume; MCH: mean corpuscular hemoglobin; MCHC: mean corpuscular hemoglobin concentration; RDW: red cell distribution width; Plt: platelet; CRP: c-reactive protein; FEU: fibrinogen equivalent units; BNP: b-type natriuretic peptide; BUN: blood urea nitrogen; AST: aspartate aminotransferase; ALT: alanine aminotransferase; PT: prothrombin time; INR: international normalized ratio; PTT: partial thromboplastin time; HPF: high power field

	Day 1	Day 3	Day 4	Day 5	Day 6	Day 7	Reference
WBC (10^9^/L)	8.92		15.86	11.9	13.45	10.45	4.50-14.50
Neutrophil (%)	53.5		80.6	82.4	73.2	73.1	
Lymphocyte (%)	16.7		13.9	12.6	15.5		
Monocyte (%)	0.9		2.6	2.2	7.3		
Eosinophil (%)	1.7		2.0	0.1	0.0		
Basophil (%)	0.0		0.1	0.1	0.1		
RBC (10^12^/L)	4.28		3.6	3.51	3.81		3.85-4.75
Hgb (g/dL)	11.3		9.3	9.1	9.9		12.0-14.5
Hct (%)	33.4		27.4	27.1	29.6		31.5-38.3
MCV (fL)	78.0		76.1	77.2	77.7		78.2-85.2
MCH (pg)	26.4		25.8	25.9	26.0		27.5-30.0
MCHC (g/dL)	33.8		33.9	33.6	33.4		34.3-35.8
RDW (%)	14.1		15.6	15.8	15.9		12.8-14.2
Plt (10^9^/L)	234		338	466	694		199-369
CRP (mg/dL)	18.0	25.3	26.3	20.1	12.8	5.2	<0.3
D-Dimer (ng/mL FEU)	1439		3919	1988	972	423	<500-558
Troponin I (pg/mL)	<3		126.4	117.1	90.8	42.1	<78.5
BNP (pg/mL)			11591	11889	7214	3721	<125
Ferritin (ng/mL)	84		214				26-388
Lactate dehydrogenase (units/L)	258						87-241
BUN (mg/dL)	10		9				7-18
Creatinine (mg/dL)	0.47	0.52	0.49				0.30-0.70
AST (units/L)	31						15-37
ALT (units/L)	26						16-61
Alkaline Phosphatase (units/L)	224						45-117
PT (s)	15.5						10.2-13.4
INR	1.3						0.9-1.1
PTT (s)	30.7						24.9-37.7
Blood Culture	No growth	No growth					No growth
Urine color		Yellow					Yellow
Urine appearance		Cloudy					Clear
Urine pH		6.0					5.0-6.0
Urine specific gravity		1.020					1.010-1.025
Urine protein		Negative					Negative
Urine glucose		Negative					Negative
Urine ketone		Negative					Negative
Urine blood		Negative					Negative
Urine nitrite		Negative					Negative
Urine bilirubin		Negative					Negative
Urine urobilinogen		Normal					normal
Urine Leukocyte Esterase		Negative					Negative
Urine RBC/HPF		2					0-2
Urine WBC/HPF		Negative					Negative
anti-SARS-CoV-2 nucleocapsid antibody			Positive				Negative
anti-SARS-CoV-2 spike protein receptor-binding domain antibody (U/mL)			25.5				<0.8

On the morning of the fourth day of his hospital course, the patient showed minimal improvement clinically, and a repeated neck CT was obtained. The size of the retropharyngeal fluid decreased to 58 x 13 x 6 mm (craniocaudal x transverse x anterior-posterior). It remained free of attenuation or enhancement in the surrounding soft tissue. Surgical intervention was not indicated. An echocardiogram was also obtained to address the concerning elevated heart rate and revealed an ejection fraction of 43%, a mild dilatation noticed in the left main coronary artery (0.3 cm, Olivieri z-score 0.71) and right coronary artery (0.4 cm, Olivieri z-score 1.69), and a moderate mitral and tricuspid valve regurgitation. Laboratory findings revealed elevated inflammatory markers (Table 2).

The findings on the echocardiogram, the skin rash, persistent fever, and elevated inflammatory markers placed the patient in the criteria for MIS-C after no source of infection could be found on other tests. He was immediately transferred to the pediatric intensive care unit (PICU) for an escalated level of care to monitor his cardiopulmonary functions continuously. Fortunately, his oxygen saturation remained about 96% on room air, and he felt comfortable breathing without difficulties. The lab for quantifying anti-SARS-CoV-2 nucleocapsid antibody and anti-SARS-CoV-2 spike protein receptor-binding domain antibody was sent (Table 2). These laboratory studies were not routinely done in our facility and were sent to an outside source. While the team was waiting for the result, we added daily laboratory studies for B-Natriuretic Peptide and Troponin I to trend the cardiac function. The initial baseline values were elevated (Table 2). 

The patient was also started on IVIG 2mg/kg infusion over 48 hours in 2 sessions to avoid volume overload from rapid infusions. Methylprednisolone 2mg/kg IV Bid was proposed to continue for five days. The patient was eventually discharged four days after starting the IV methylprednisolone, and the steroid for the fifth day was switched to oral prednisone and administered at home. Per institution protocol, the patient was also tested for MRSA colonization of nares before being transferred to the PICU. Vancomycin and Ceftriaxone were continued until MRSA came back negative. The patient spiked a fever to 102.7 degrees Fahrenheit immediately after the first session of IVIG infusion, which was expected. Since then, he has remained afebrile (Figure 1). Clinically, he felt much better, and the neck pain improved significantly. A repeated laboratory study showed down-trending D-dimer, C-Reactive Protein, and WBC count. In the afternoon of Day 5, both anti-SARS-CoV-2 nucleocapsid and anti-SARS-CoV-2 spike protein receptor-binding domain antibodies returned positive (Table 2), meaning the COVID-19 virus infected the patient. However, he probably was asymptomatic since neither his parents nor himself could recall having any COVID symptoms for the past few months. The significant lab findings on the COVID-19 antibody completed the final piece of the puzzle of his clinical picture. A repeated echocardiogram on Day 6 of his hospital course showed an improved ejection fraction at 50%, mild but improved coronary arteries dilation, noticed in the left main coronary artery (0.28 cm, Olivieri z-score 0.36), and right coronary artery (0.35 cm, Olivieri z-score 0.90) and mild mitral valve regurgitation. The regurgitation from the tricuspid valve had resolved. On Day 7 of his hospital course, he improved clinically, evidenced by a normal white count, down-trended D-Dimer, C-reactive protein, Troponin I, and B-Natriuretic Peptide (Table 2). The patient's MRSA result was negative, and Vancomycin was stopped. A CT was repeated to assess the retropharyngeal fluid collection and showed a measurement of 41 x 7 x 3 mm (craniocaudal x transverse x anterior-posterior), which continued to decrease compared to the result from the previous CT 4 days ago. The otolaryngologist recommended treating the fluid collection as a retropharyngeal phlegmon with oral clindamycin or IV ceftriaxone and following up as an outpatient visit in 2 weeks. The patient was discharged on oral prednisone 2.5mg/kg for one day to complete the proposed 5-day treatment of steroid, followed by a schedule for oral prednisolone taper over two weeks and outpatient follow-up with pediatric cardiology after the taper is done. 

## Discussion

The total number of MIS-C patients meeting the case definition reported to CDC is 7459 as of March 1, 2022 [3]. According to the CDC, MIS-C is prevalent among non-Hispanic white, non-Hispanic black, and Hispanic patients, with Hispanic and non-Hispanic Black populations disproportionately affected by COVID-19 [3]. In terms of sex, male patients are prone to MIS-C, representing 60.49% of the total cases. Age distribution mimics a normal distribution "bell curve" with the mean (the highest point) coinciding with the age group of 5-11-year-olds [3]. The mean age for MIS-C is 9 [4].

Currently being considered a late manifestation of COVID-19 infection, MIS-C's highest risk factor remains exposure to the COVID-19 virus [3]. As in our case patient, even though the patient's family could not recollect any memory of COVID symptoms or COVID exposures, the patient tested positive for both the anti-SARS-CoV-2 nucleocapsid antibody and anti-SARS-CoV-2 spike protein receptor-binding domain antibody. The testing assay for the anti-SARS-CoV-2 nucleocapsid antibody will not detect antibodies induced by the currently available COVID-19 vaccines. In other words, A positive result for the anti-SARS-CoV-2 nucleocapsid antibody can be used to confirm a recent or prior COVID-19 infection [5]. 

Surprisingly, MIS-C is more prevalent among asymptomatic children with COVID-19 who are positive for RTPCR (reverse transcriptase-polymerase chain reaction) or positive antibodies to SARS-COV-2 (Severe Acute Respiratory Syndrome Coronavirus). The positive results of the diagnostic tests suggest that these children were in the convalescent phase of COVID-19 infection when they developed MIS-C [6]. Because respiratory symptoms are not common among patients with MIS-C, their manifestations were initially thought to be a variant of Kawasaki disease. They share similar features, such as coronary artery dilations, skin rashes, and systemic inflammations. However, Kawasaki disease is common in children of Asian ancestry, while MIS-C is common in children of African ancestry, indicating a racial predisposition [7]. Another feature that separates MIS-C from Kawasaki disease is that the cardiac symptoms in MIS-C are milder than that of Kawasaki disease. Most children with MIS-C can regain cardiac functions to full capacities after treatment, as seen in the index case.

Cardiac manifestations occur commonly in children with MIS-C, estimated in up to 67-80% of cases, and often include coronary artery aneurysm and ventricular dysfunction [8-10]. Coronary artery aneurysms are common in male patients with mucocutaneous and conjunctival involvement [11]. The degree of the coronary artery dilation is often mild and able to normalize within 30 days [12]. The etiology is currently unclear and could be secondary to generalized inflammation. Ventricular dysfunction is characterized by a decreased ejection fraction illustrated by an echocardiogram and is the most reported finding, occurring in up to 50% of children with MIS-C [13,14]. B-type natriuretic peptide (BNP) or pro-BNP values are the frequently used markers for describing ventricular dysfunction in children with MIS-C [12]. Similar to coronary artery aneurysm, the etiology of ventricular dysfunction is currently unclear; potential candidates include acute myocarditis, hypoxic injury secondary to coronary artery disease, and systemic inflammatory response syndrome [15-17]. Our index patient had a normal ECG test during admission with normal rate and sinus rhythm. His cardiac involvement was not unveiled until four days later by an echocardiogram, granted by a persistently elevated heart rate. Due to the high frequency of cardiac involvement in children with MIS-C, cardiac workups, including but not limited to ECG, echocardiogram, and cardiac enzymes and markers, should be conducted more regularly for pediatric patients with suspicions of MIS-C [18].

Our case report's most important differential diagnostic dilemma was the retropharyngeal phlegmon in the raging COVID pandemic. Interestingly, it seems that many clinicians around the world have questioned the link between MIS-C and retropharyngeal phlegmon. One study suggests that 28% of patients diagnosed with MIS-C at their institution had neck complaints due to retropharyngeal edema [19]. Even though most of these patients received antimicrobials in addition to anti-inflammatory treatment, retrospective reviews suggested that the retropharyngeal edema was inflammatory rather than infectious [19]. It is worth noting that otolaryngologic manifestations such as retropharyngeal edema may often be overlooked during the exclusionary process of MIS-C diagnosis and not be considered one of the organ systems with inflammation. More systematic studies are needed to confirm the possible association between MIS-C and retropharyngeal edema. Such confirmation will help guide the treatment of retropharyngeal edema in the clinical context of MIS-C by avoiding over-treatment with unnecessary antibiotics and help diagnose MIS-C if retropharyngeal edema is considered a characteristic feature of the systemic inflammation involving multiple organ systems [20].

## Conclusions

MIS-C is currently considered a late manifestation of COVID-19 infection, and it is an exclusionary diagnosis. MIS-C should be suspected in pediatric patients presenting with persistent fevers and generalized lethargy even without known exposure to the COVID-19 virus. MIS-C is more prevalent in asymptomatic COVID-19 patients. Frequently, MIS-C involves cardiac manifestation, and cardiac workups are essential for diagnosing MIS-C. Last but not least, the retropharyngeal edema that the case patient presented with was thought to be coincidental; however, this case report reveals that the reason for sore throat in MIS-C might be retropharyngeal edema and helps to diagnose suspected MIS-C patients with pending confirmatory tests of COVID-19. More studies are needed to confirm the association between retropharyngeal edema as a presenting symptom and MIS-C and guide the treatments of retropharyngeal edema in MIS-C.
